# Association between sleep patterns and galectin-3 in a Chinese community population

**DOI:** 10.1186/s12889-024-18811-4

**Published:** 2024-05-16

**Authors:** Lin Liu, Juanying Zhen, Shuyun Liu, Lijie Ren, Guoru Zhao, Jianguo Liang, Aimin Xu, Chao Li, Jun Wu, Bernard Man Yung Cheung

**Affiliations:** 1 Department of Medicine, School of Clinical Medicine, The University of Hong Kong, Queen Mary Hospital, Hong Kong SAR, China; 2 Department of Neurology, Peking University Shenzhen Hospital, Shenzhen, China; 3 Department of Neurology, Shenzhen Longhua District Central Hospital, Shenzhen, China; 4 Department of Neurology, The First Affiliated Hospital of Shenzhen University, Shenzhen Second People’s Hospital, Shenzhen, China; 5 CAS Key Laboratory of Human-Machine Intelligence-Synergy Systems, Research Center for Neural Engineering, Shenzhen Institute of Advanced Technology, Chinese Academy of Sciences, Shenzhen, China; 6 Precision Health Research Center Company Limited, Hong Kong SAR, China; 7 State Key Laboratory of Pharmaceutical Biotechnology, The University of Hong Kong, Pokfulam, Hong Kong SAR, China; 8 Institute of Cardiovascular Science and Medicine, The University of Hong Kong, Pokfulam, Hong Kong SAR, China

**Keywords:** Inflammation, Galectin-3, Sleep disturbance, Sleep duration, Napping duration

## Abstract

**Background:**

Irregular sleep patterns have been associated with inflammation. Galectin-3, a novel biomarker, plays an important role in inflammation. We investigated the relationship between sleep patterns and galectin-3 in a Chinese population.

**Methods:**

A total of 1,058 participants from the Shenzhen-Hong Kong United Network on Cardiovascular Disease study were included in the analysis. Age and sex-adjusted linear regression models were employed to investigate the relationship between galectin-3 level and traditional metabolic biomarkers. Logistic regression models were used to estimate the association among sleep disturbance, nighttime sleep duration, and daytime napping duration and elevated galectin-3, with elevated galectin-3 defined as galectin-3 level > 65.1 ng/ml.

**Results:**

Of study participants, the mean age was 45.3 years and 54.3% were women. Waist circumference, natural logarithm (ln)-transformed triglyceride, and ln-transformed high sensitivity C-reactive protein were positively associated with galectin-3 level (age and sex-adjusted standardized β [95% confidence interval (CI)], 0.12 [0.04, 0.21], 0.11 [0.05, 0.17], and 0.08 [0.02, 0.14], respectively). Sleep disturbance was associated with elevated galectin-3 (odds ratio [95% CI], 1.68 [1.05, 2.68], compared to those without sleep disturbance) after adjusting for traditional metabolic biomarkers. No interaction was observed between galectin-3 and age, sex, obesity, hypertension, and diabetes on sleep disturbance. No association was found between nighttime sleep duration or daytime napping duration and elevated galectin-3.

**Conclusions:**

Our study provides evidence of a significant association between sleep disturbance and elevated galectin-3 level, independent of traditional metabolic biomarkers. Screening and interventions on galectin-3 could assist in preventing sleep disturbance-induced inflammatory disease.

**Supplementary Information:**

The online version contains supplementary material available at 10.1186/s12889-024-18811-4.

## Introduction

Adequate sleep duration and high-quality sleep play a pivotal role in fostering optimal physical, mental, and emotional functioning [1]. Sleep primarily influences two key effector systems: the hypothalamus-pituitary-adrenal (HPA) axis and the sympathetic nervous system (SNS), which subsequently regulate immune responses [2]. Sleep disturbances can trigger immune responses by activating the SNS pathway, this activation upregulates the transcription of proinflammatory immune response genes such as interleukin (IL)1B, tumor necrosis factor (TNF), and IL6, leading to a heightened systemic inflammatory activity [3, 4]. Population studies have consistently shown a positive correlation between sleep disturbance and inflammatory biomarkers. Individuals experiencing poor sleep quality tend to exhibit elevated levels of C-reactive protein (CRP) and IL-6 [5, 6]. Moreover, heightened systemic inflammation contributes to the development of a range of adverse health outcomes. For instance, inflammation impairs the endothelial cells lining the heart and blood vessels, leading to atherosclerosis and heart disease [7]. Furthermore, peripheral cytokines can reach the brain through blood–brain barrier disruption or penetration of peripheral immune cells, thus contributing to the process of neurodegeneration [8–10]. It is plausible that inflammation significantly contributes to the intricate relationship linking sleep deprivation to cardiovascular diseases, metabolic disorders, and neurodegenerative conditions [11, 12].

Galectin-3, a β-galactoside-binding lectin, is highly expressed and secreted from macrophages and functions as a regulatory protein in different stages of inflammation and tissue fibrinogenesis [13]. By influencing these fundamental cellular mechanisms, galectin-3 may have a broader impact on the inflammatory response and tissue remodeling following sleep disturbances. Recent research studies have established a connection between galectin-3 and both peripheral information and neuroinflammation, with positive associations noted between galectin-3 and cardiovascular as well as neurodegenerative diseases [14, 15]. Galectin-3 has been identified as a central upstream regulator of the immune response in Alzheimer’s disease [16]. It has also been observed that galectin-3 levels increase significantly in patients with Obstructive Sleep Apnea (OSA), and this increase further promotes neuroinflammation and oxidative stress via regulating NLRP3 [17]. Moreover, galectin-3 inhibitors have been shown to alleviate diabetes-associated cognitive impairment by reducing oxidative stress and neuroinflammation both in vivo and in vitro [18], which confirms the important role of galectin-3 in systemic inflammation. However, the relationship between galectin-3 and sleep patterns in the general population has not yet been investigated.

Therefore, this study hypothesizes that elevated galectin-3 levels may result from abnormal sleep patterns and further associate with inflammation-driven diseases. The objectives of this research are twofold: (1) to explore the relationship between sleep patterns and circulating levels of galectin-3 in a Chinese community population; and (2) to enhance understanding of the complex interplay among sleep, inflammation, and chronic disease.

## Materials and methods

### Study population

The Shenzhen-Hong Kong United Network on Cardiovascular Disease (SHUN-CVD) study is an ongoing population-based study initiated in late 2020 [19]. In total, two study waves encompassing 3,400 participants from communities in Shenzhen were enrolled and underwent face-to-face interviews, physical examinations, and laboratory tests. In the present study, we included 1,060 participants from the initial wave and, after excluding those without valid data on serum galectin-3 levels (*N* = 2), a total of 1,058 participants were included. The study was reviewed and approved by the institutional review boards of Peking University Shenzhen Hospital (approval number [2021] No.040) and the University of Hong Kong (IRB Ref.: UW 20–410). All enrolled participants provided written informed consent.

### Measurement and definition

Structured questionnaires were employed to collect demographic data, medical history, medication use, lifestyle and sleep patterns. Trained examiners conducted physical examinations, including systolic blood pressure (SBP), diastolic blood pressure (DBP), body weight and height and waist circumference (WC). Blood samples were collected after a minimum 8-hour fasting period for fasting blood glucose (FBG), hemoglobin A1c (HbA1c), high-sensitivity C-reactive protein (Hs-CRP), total cholesterol, triglyceride, low-density lipid (LDL) cholesterol, and high-density lipid (HDL) cholesterol. Galectin-3 level were measured using ELISA kits supplied by ImmunoDiagnostics Limited. Two samples of known concentration were test for Intra-assay and Inter-assay Precision, respectively. The intra-assay coefficients of variation (CVs) were 4.5% and 6.2%, and the inter-assay CVs were 5.5% and 6.1%. Detailed procedures can be found on the website listed here: https://www.immunodiagnostics.com.hk/product-page/human-galectin-3-elisa-kit.

Current smoking was defined as the daily use of any tobacco product. Participants were considered as drinkers if they consumed any type of alcoholic beverage at least once a week. Body mass index (BMI, kg/m^2^) was calculated as weight (in kilograms) divided by height (in meters squared). BMI ≥ 28 kg/m^2^ was defined as obesity according to the criteria recommended for Chinese adults. Abdominal obesity was defined as a WC ≥ 90 cm in males or a WC ≥ 85 cm in females. Hypertension was defined as SBP ≥ 140 mm Hg or DBP ≥ 90 mm Hg or the use of antihypertensive medication. Diabetes was defined as fasting blood glucose ≥ 126 mg/dL or HbA1c ≥ 6.5% or the use of antidiabetic medication. OSA was defined as self-reported medical history.

Three aspects of sleep patterns were assessed: sleep disturbance, nighttime sleep duration and daytime napping duration. Sleep disturbance was defined as self-reported poor sleep quality (response “bad” or “very bad” to the question “How do you rate your sleep quality?“) and/or difficulty falling asleep and/or frequently waking up during the night. We used 7 h and 60 min as the cutoffs for nighttime sleep duration and daytime napping duration, respectively [20, 21].

Clinical cutoffs for galectin-3 have not yet been established. To identify participants with elevated galectin-3, we chose a cutoff that corresponded to a similar percentage as elevated Hs-CRP (Hs-CRP > 3 mg/L [*n* = 89, 8.4%]), thus elevated galectin-3 indicates a galectin-3 level > 65.1 ng/mL (*n* = 89 [8.4%]), such an approach have been used in previous study [22]. Additionally, since there is no specific cutoff for galectin-3 in clinical practice, we conducted a sensitivity analysis using the 50%, 60%, 70%, 80%, and 90% quantiles as alternative cutoffs to define elevated galectin-3 levels.

### Statistical analysis

Continuous values were reported as means ± standard deviation (SD) or median [Q1, Q3] and compared using the Kruskal-Wallis test or the one-way analysis of variance. Categorical variables were reported as counts and percentages and compared by the χ2 test. The sex-specific distribution of galectin-3 is shown in Figure S1, with a similar and normal distribution among sexes. The relationship between galectin-3 levels and traditional metabolic biomarkers (SBP, DBP, BMI, WC, FBG, TG, HDL cholesterol, and Hs-CRP) was tested using Pearson correlation. Univariate linear regression and Multivariate linear regression (adjusted for age and sex) were further employed to estimate the relationship between galectin-3 level and traditional metabolic biomarkers. Logistic regression was utilized to investigate the effect of elevated galectin-3 on sleep disturbance (participants without sleep disturbance as the reference group), nighttime sleep duration (≥ 7 h as the reference group) and daytime napping duration (< 60 min as the reference group). Three models were fitted: model 1 was the crude model, model 2 adjusted for age and sex, and model 3 adjusted for age, sex, current smoking, alcohol consumption, OSA, SBP, BMI, WC, FBG, TG, HDL-cholesterol, and Hs-CRP. Subgroup analyses were performed by age (< 50, ≥ 50 years), sex (men, women), obesity (yes, no), abdominal obesity (yes, no), hypertension (yes, no), and diabetes (yes, no). To test for modifications and interactions, likelihood-ratio tests were used. Sensitivity analysis was conducted to investigate the relationship between sleep disturbance subtypes and elevated galectin-3. We further conducted sensitivity analysis using 8 h and 30 min as the cutoffs for nighttime sleep duration and daytime napping duration, respectively. Three logistic models mentioned above were fitted. All analyses were conducted using R 4.2.1. A p-value < 0.05 was considered statistically significant for all analyses.

## Results

Table 1 summarizes the baseline characteristics of the study population. A total of 1,058 participants with a mean age of 45.3 ± 10.3 years were included in the study, of which 575 (54.3%) were female. Compared with participants who had normal galectin-3 level, participants with elevated galectin-3 level were older and had higher SBP, WC, Hs-CRP, total cholesterol, and were more likely to have hypertension. Table S1 shows the baseline characteristics according to sleep patterns. Compared with participants without sleep disturbance, participants with sleep disturbance were more likely to be female, had lower HbA1c and higher galectin-3 level. Participants with nighttime sleep duration < 7 h were older and had higher level of total cholesterol and LDL-cholesterol, compared to those ≥ 7 h. Participants with daytime napping duration ≥ 60 min had a higher prevalence of diabetes, compared to those < 60 min.

**Table 1:** Baseline characteristic of the study population

	Overall	Galectin-3 level, ng /ml		*P*-value
		Normal	Elevated	
	1058	969	89	
Age, year	45.29 ± 10.25	44.69 ± 9.87	51.84 ± 11.89	< 0.001
Female (%)	575 (54.3)	522 (53.9)	53 (59.6)	0.358
Current smoker (%)	102 (9.6)	95 (9.8)	7 (7.9)	0.685
Alcohol drinker (%)	53 (5.0)	50 (5.2)	3 (3.4)	0.627
Systolic BP, mmHg	119.28 ± 16.24	118.95 ± 16.16	122.89 ± 16.82	0.03
Diastolic BP, mmHg	78.68 ± 10.49	78.63 ± 10.49	79.25 ± 10.58	0.596
Body mass index, kg/m²	23.67 ± 3.58	23.65 ± 3.61	23.97 ± 3.29	0.426
Waist, cm	89.81 ± 15.28	89.42 ± 15.14	93.97 ± 16.22	0.007
Fasting blood glucose, mmol/L	4.65 ± 1.14	4.64 ± 1.15	4.81 ± 0.91	0.177
HbA1c, %	5.80 ± 0.73	5.79 ± 0.74	5.87 ± 0.60	0.334
Hs-CRP, mg/L	0.77 [0.45, 1.41]	0.74 [0.44, 1.34]	1.07 [0.60, 1.92]	< 0.001
Galectin-3, ng/mL	51.26 ± 21.41	47.54 ± 7.97	91.75 ± 54.77	< 0.001
Total cholesterol, mmol/L	4.92 ± 0.89	4.90 ± 0.88	5.10 ± 0.92	0.043
Triglyceride, mmol/L	1.14 [0.81, 1.68]	1.13 [0.80, 1.67]	1.29 [0.86, 2.04]	0.026
HDL-Cholesterol, mmol/L	1.38 ± 0.30	1.37 ± 0.29	1.44 ± 0.40	0.053
LDL-Cholesterol, mmol/L	2.72 ± 0.73	2.72 ± 0.73	2.75 ± 0.74	0.783
Hypertension (%)	229 (21.7)	200 (20.7)	29 (33.0)	0.011
Diabetes (%)	81 (7.8)	73 (7.7)	8 (9.1)	0.806
Obstructive Sleep Apnea (%)	16 (1.5)	15 (1.5)	1 (1.1)	0.999
Sleep disturbance (%)	348 (32.9)	310 (32.0)	38 (42.7)	0.052
Nighttime sleep < 7 h (%)	301 (28.4)	273 (28.2)	28 (31.5)	0.593
Daytime napping > 1 h (%)	128 (12.1)	115 (11.9)	13 (14.6)	0.556

Elevated galectin-3 was defined as galectin-3 level > 65.1 ng/ml.

Continuous variables are expressed as mean ± standard deviation or as median [IQR].

Categorical variables are expressed as number (percent).

HDL, high-density lipid. LDL, low-density lipid. Hs-CRP, high sensitivity C- reactive protein. HbA1c, hemoglobin A1c

**Fig. 1:**
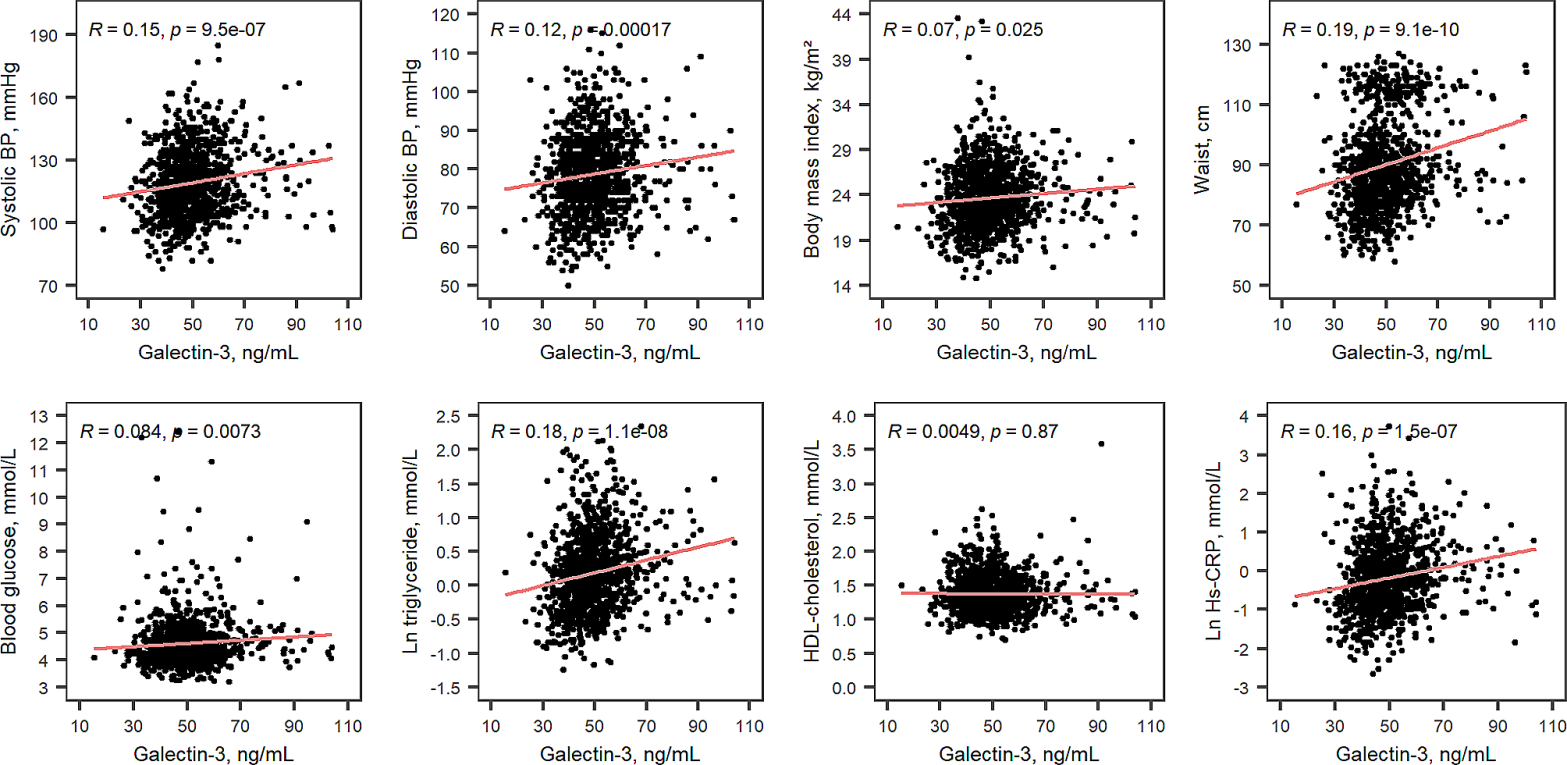
Correlations of galectin-3 level and metabolic biomarkers BP, blood pressure. Ln, log-transformed. HDL, high-density lipid. LDL, low-density lipid. Hs-CRP, high sensitivity C- reactive protein

The Pearson correlations between galectin-3 level and traditional metabolic biomarkers are shown in Fig. 1. Galectin-3 level was positively correlated with SBP (*R* = 0.15, *P* < 0.001), DBP (*R* = 0.12, *P* < 0.001), BMI (*R* = 0.07, *P* = 0.03), WC (*R* = 0.19, *P* < 0.001), FBG (*R* = 0.08, *P* = 0.007), ln-transformed triglyceride (*R* = 0.18, *P* < 0.001) and ln-transformed Hs-CRP (*R* = 0.16, *P* < 0.001). The linear regression between galectin-3 levels and traditional metabolic biomarkers are shown in Table 2. After adjusting for age and sex, WC, ln-transformed triglyceride and ln-transformed Hs-CRP were positively associated with galectin-3 level [Standardized β (95% Confidence Interval), 0.12 (0.04, 0.21), 0.11 (0.05, 0.17), and 0.08 (0.02, 0.14), respectively].

**Table 2:** Association between galectin level and metabolic markers

	Unadjusted model		Adjusted model	
	β (95%CI) ^b^	P-value	β (95%CI) ^b^	P-value
Systolic BP, mmHg	0.12 (0.04, 0.19)	0.003	0.04 (-0.04, 0.12)	0.33
Diastolic BP, mmHg	0.06 (-0.02, 0.13)	0.15	0.01 (-0.07, 0.09)	0.83
Body mass index, kg/m²	0.06 (0, 0.12)	0.05	0.04 (-0.02, 0.1)	0.15
Waist, cm	0.16 (0.07, 0.24)	< 0.001	0.12 (0.04, 0.21)	0.005
Fasting blood glucose, mmol/L	0.05 (-0.02, 0.12)	0.15	0.01 (-0.06, 0.08)	0.77
Triglyceride, mmol/L ^a^	0.12 (0.06, 0.18)	< 0.001	0.11 (0.05, 0.17)	< 0.001
HDL-Cholesterol, mmol/L	0.06 (-0.02, 0.13)	0.14	0.06 (-0.02, 0.14)	0.13
Hs-CRP, mg/L ^a^	0.1 (0.04, 0.16)	< 0.001	0.08 (0.02, 0.14)	0.01

CI, confidence interval. BP, blood pressure. HDL, high density lipid. Hs-CRP, high-sensitivity C-reaction protein

^a^ Log-transformed (natural logarithm)

^b^ Standardized estimated regression coefficients

Adjusted model: adjusted for age and sex

The association between elevated galectin-3 and sleep patterns are presented in Table 3. Sleep disturbance was significantly associated with elevated galectin-3 in the fully adjusted model [Odds ratio (95% Confidence Interval), 1.68 (1.05, 2.68)]. However, neither nighttime sleep duration nor daytime napping duration was associated with elevated galectin-3 [[Odds ratio (95% Confidence Interval), 1.06 (0.65, 1.73)] for long nighttime sleep duration and 1.08 (0.56, 2.09) for short daytime napping duration]. The subgroup analysis in Table S2 shows that there was no interaction of sleep disturbance with age, sex, obesity, abdominal obesity, hypertension and diabetes on elevated galectin-3 (all P-values for interaction > 0.05).

**Table 3:** Association between sleep patterns and elevated galectin-3

	Odd ratio (95% confidence interval)		
	Sleep disturbance ^a^	Nighttime sleep duration ^b^	Daytime napping duration ^c^
Model 1	1.58 (1.02, 2.46)	1.17 (0.73, 1.87)	1.27 (0.68, 2.36)
Model 2	1.56 (1.00, 2.46)	1.00 (0.62, 1.62)	1.08 (0.56, 2.06)
Model 3	1.68 (1.05, 2.68)	1.06 (0.65, 1.73)	1.08 (0.56, 2.09)

^a^ yes vs. no. ^b^ < 7 hours vs. ≥ 7 hours. ^c^ ≥ 60 minutes vs. < 60 minutes

Model 1. crude model

Model 2. adjusted for age and sex

Model 3. adjusted for age, sex, current smoking, alcohol consumption, systolic blood pressure, Obstructive Sleep Apnea, body mass index, waist, fasting blood glucose, triglyceride, high density lipid cholesterol and high-sensitivity C- reactive protein

Elevated galectin-3 was defined as galectin-3 level > 65.1 ng/ml

Sensitivity analysis on the relationship between sleep patterns and elevated galectin-3 levels using different cutoff values was presented in Table S3. The analysis revealed that sleep disturbance was consistently and significantly associated with elevated galectin-3 levels across the various cutoffs tested, indicating the robustness of the findings observed in the main analysis. Table S4 shows the relationship between sleep disturbance subtypes and elevated galectin-3. In the fully adjusted models, self-reported poor sleep quality and difficulty in falling asleep were significantly associated with elevated galectin-3[OR (95%CI), 1.33 (1.02, 1.74) and 1.93 (1.06, 3.51), respectively], while frequently waking up during the night was not associated with elevated galectin-3 [OR (95%CI), 1.40 (0.86, 2.27)]. Table S5 presents the relationship between elevated galectin-3 and nighttime sleep duration and daytime napping duration using different cutoffs. The results keep unchanged with the main analysis.

## Discussion

In this population-based study, we investigated the relationship among sleep patterns, galectin-3 level and traditional metabolic biomarkers. We found a significant association between sleep disturbances and elevated galectin-3 level. However, no associations were observed between nighttime sleep duration or daytime napping duration and galectin-3 level.

A key finding in our study is the significant associations between elevated galectin-3 level and traditional metabolic biomarkers. This finding aligns with previous literature suggesting that galectin-3 plays a significant role in the pathophysiology of cardiometabolic diseases [23]. More precisely, our analysis demonstrated a positive correlation between galectin-3 levels and TG, BMI, and Hs-CRP, after adjusting for age and sex confounders. Galectin-3 has been found to promote the differentiation and proliferation of adipocytes [24], and during this process, both the mRNA and protein expression levels of galectin-3 increase in a time-dependent manner [25]. This process leads to the accumulation of body fat, eventually resulting in conditions such as obesity and hypertriglyceridemia [26]. The positive relationship between galectin-3 and Hs-CRP appears more multifaceted. On the one hand, galectin-3 and Hs-CRP can initially rise in response to immunoreaction [13, 27], on the other hand, galectin-3 can further serve as triggers or amplifiers of inflammatory reactions, thereby promoting cytokine secretion [28, 29].

Our research indicates a robust association between sleep disturbances and elevated levels of galectin-3. Notably, sleep disturbances and inflammation maintain a bidirectional relationship, each with the potential to influence the other. Sleep serves as a critical restorative process for the body, integral to the regulation of the immune system [1]. Sleep deprivation may not only stimulate peripheral inflammation by increasing the secretion of pro-inflammatory cytokines, such as IL-6 and TNF [3], but it can also contribute to neuroinflammation. Both acute and chronic experimental sleep deprivation in animals and humans leads to the accumulation of brain beta-amyloid (Aβ) and plaque formation [30, 31], which in turn triggers the activation of immune cells in the brain, such as microglia and astrocytes [32]. Furthermore, a recent animal study suggested that galectin-3 promotes Aβ oligomerization and Aβ toxicity in a mouse model [33], underscoring galectin-3’s role in sleep disturbance-induced inflammation. Conversely, inflammation can adversely affect sleep. Certain cytokine-associated substances, including TNF and IL-1 soluble receptors, can function as antagonists, inhibiting spontaneous sleep and causing symptoms such as insomnia or excessive daytime sleepiness [34]. Additionally, pro-inflammatory cytokines and galectin-3, acting as signaling molecules on neurotransmitter metabolism [35, 36], can cause neurotransmitter dysregulation and impair the sleep modulatory system [37]. Given the reciprocal relationship between sleep disturbances and inflammation, our study points to the potential use of galectin-3 inhibitors to alleviate inflammatory processes and subsequently improve sleep quality.

It is important to note that OSA serves as a significant risk factor for cardiometabolic diseases [38]. Several mechanisms have been described to explain the association between OSA and cardiometabolic diseases, with inflammation being the most crucial one [39, 40]. Previous studies have identified higher levels of galectin-3 in OSA patients, and these levels have been found to correlate with the severity of OSA [41]. Repetitive hypoxia and reoxygenation during the night in OSA promote oxidative stress by increased production of reactive oxygen species and angiogenesis and enhanced sympathetic activation [42]. Galectin-3 has been identified as a novel regulator of oxidative stress, and inhibiting this protein has been shown to restore the antioxidant peroxiredoxin-4 and suppress oxidative stress [43]. In the context of our study, only 1.5% of the participants reported a history of OSA, which is lower than what the existing literature suggests [44]. This discrepancy is likely due to the underrecognition and underreporting of OSA symptoms by patients, as well as the fact that our study population is relatively healthy. The average age of the study population was 45 years, with fewer risk factors (low smoking prevalence and relatively lower BMI) [41]. We found no difference in the prevalence of OSA between the different galectin-3 groups, which may be attributed to the different cutoff for galectin-3 compared to previous studies and the lack of polysomnography or other objective sleep studies in our methodology to confirm diagnoses [17]. Given the high prevalence of undiagnosed OSA, it is conceivable that some of the observed associations between sleep disturbance and galectin-3 may be attributable to undetected OSA cases within our cohort. To address this limitation, we conducted additional analyses that adjusted for the known risk factors for OSA, as previously mentioned. While this statistical approach cannot replace objective sleep assessments, it can mitigate some of the potential confounding effects. Despite these adjustments, the association between sleep disturbance and galectin-3 levels remained significant. This finding reinforces the notion that sleep disturbance in the general population, albeit not as severe as OSA, can still lead to an increase in galectin-3 levels. However, the precise mechanisms linking sleep disturbance to elevated galectin-3 levels require further investigation.

Interestingly, the duration of either daytime naps or nighttime sleep was not associated with galectin-3 level. We further found the association between self-reported poor sleep quality and elevated galectin-3 level. This finding suggests that galectin-3 level was influenced by sleep quality rather than sleep duration. The relationship between nighttime sleep duration and inflammatory biomarkers remains unclear. A recent meta-analysis indicated that short sleep duration was not associated with (CRP), IL-6, or tumor necrosis factor α [45]. Similarly, the association between daytime napping duration and inflammatory biomarkers varies across populations. No association was found between daytime napping duration and Hs-CRP in a Chinese population [46], while a positive association between daytime napping and CRP was observed in a U.S young adults [21].

### Strengths and limitation

This study has several strengths, including the large population-based sample and the adjustment for potential confounders (age, sex, OSA, and traditional metabolic biomarkers). However, there are limitations to be considered. First, the cross-sectional design limits our ability to establish causality between sleep disturbances and elevated galectin-3 level. Second, sleep patterns and OSA were assessed using self-reported questionnaires, which may be subject to recall bias and misclassification. Objective sleep measurements and the well-validated questionnaire, such as polysomnography and Pittsburgh sleep quality index (PSQI), could provide more accurate assessments. Lastly, we did not consider other potential confounding factors, such as medication use or comorbidities, which may influence sleep patterns and galectin-3 level.

## Conclusion

In conclusion, our findings suggest a positive correlation between galectin-3 and traditional metabolic biomarkers, as well as an association between elevated galectin-3 and sleep disturbances. Galectin-3 inhibitor could be a potential therapeutic strategy for sleep disturbance induced inflammation.

### Electronic supplementary material

Below is the link to the electronic supplementary material.

Supplementary Material 1

## Data Availability

The data that support the findings of this study are available from the corresponding author, upon reasonable request.
