# Supplementary material for: Association between sleep patterns and galectin-3 in a Chinese community population
Source: BMC Public Health. 2024 May 16;24:1323. doi: 10.1186/s12889-024-18811-4 (PMC11097462; doi:10.1186/s12889-024-18811-4)
Supplement: Supplementary file 1 — Supplementary Material 1 [file 12889_2024_18811_MOESM1_ESM.docx]

**Table S1. Baseline characteristic of the study participants according to sleep patterns**

|  | Sleep disturbance | | |  | Nighttime sleep duration | | |  | Daytime napping duration | | |
| --- | --- | --- | --- | --- | --- | --- | --- | --- | --- | --- | --- |
|  | Without | With | P-value |  | ≥ 7 hours | < 7 hours | P-value |  | < 60 minutes | ≥ 60 minutes | P-value |
|  | 710 | 348 |  |  | 757 | 301 |  |  | 930 | 128 |  |
| Age, year | 44.99 ± 10.58 | 45.89 ± 9.51 | 0.183 |  | 44.54 ± 10.21 | 47.18 ± 10.11 | <0.001 |  | 45.14 ± 9.96 | 46.36 ± 12.14 | 0.207 |
| Female (%) | 370 (52.1) | 205 (58.9) | 0.043 |  | 410 (54.2) | 165 (54.8) | 0.901 |  | 495 (53.2) | 80 (62.5) | 0.06 |
| Current smoker (%) | 75 (10.6) | 27 (7.8) | 0.18 |  | 78 (10.3) | 24 (8.0) | 0.297 |  | 91 (9.8) | 11 (8.6) | 0.788 |
| Alcohol drinker (%) | 34 (4.8) | 19 (5.5) | 0.749 |  | 33 (4.4) | 20 (6.6) | 0.167 |  | 47 (5.1) | 6 (4.7) | 1 |
| Systolic BP, mmHg | 119.84 ± 16.67 | 118.13 ± 15.29 | 0.111 |  | 119.02 ± 16.53 | 119.95 ± 15.49 | 0.401 |  | 119.28 ± 16.47 | 119.26 ± 14.57 | 0.987 |
| Diastolic BP, mmHg | 79.02 ± 10.69 | 77.99 ± 10.06 | 0.134 |  | 78.48 ± 10.54 | 79.18 ± 10.38 | 0.329 |  | 78.85 ± 10.65 | 77.48 ± 9.23 | 0.168 |
| Body mass index, kg/m² | 23.74 ± 3.47 | 23.54 ± 3.79 | 0.392 |  | 23.70 ± 3.58 | 23.60 ± 3.60 | 0.67 |  | 23.64 ± 3.41 | 23.93 ± 4.65 | 0.38 |
| Waist, cm | 89.81 ± 14.87 | 89.81 ± 16.10 | 0.999 |  | 89.53 ± 15.21 | 90.52 ± 15.44 | 0.343 |  | 89.83 ± 15.36 | 89.61 ± 14.69 | 0.879 |
| Fasting blood glucose, mmol/L | 4.69 ± 1.24 | 4.59 ± 0.89 | 0.184 |  | 4.63 ± 1.16 | 4.70 ± 1.06 | 0.375 |  | 4.64 ± 1.15 | 4.71 ± 1.00 | 0.52 |
| HbA1c, % | 5.83 ± 0.80 | 5.74 ± 0.58 | 0.043 |  | 5.78 ± 0.73 | 5.86 ± 0.73 | 0.109 |  | 5.79 ± 0.71 | 5.89 ± 0.87 | 0.161 |
| Hs-CRP, mg/L | 0.77 [0.45, 1.37] | 0.75 [0.41, 1.43] | 0.434 |  | 0.75 [0.45, 1.33] | 0.83 [0.46, 1.62] | 0.149 |  | 0.74 [0.44, 1.38] | 0.89 [0.52, 1.60] | 0.076 |
| Galectin-3, ng/mL | 50.36 ± 18.65 | 53.11 ± 26.09 | 0.049 |  | 50.98 ± 21.94 | 51.98 ± 20.04 | 0.492 |  | 51.00 ± 21.97 | 53.15 ± 16.82 | 0.288 |
| Total cholesterol, mmol/L | 4.93 ± 0.90 | 4.88 ± 0.87 | 0.361 |  | 4.87 ± 0.89 | 5.03 ± 0.87 | 0.01 |  | 4.90 ± 0.89 | 5.04 ± 0.89 | 0.095 |
| Triglyceride, mmol/L | 1.14 [0.80, 1.68] | 1.13 [0.82, 1.68] | 0.989 |  | 1.13 [0.80, 1.65] | 1.17 [0.83, 1.73] | 0.347 |  | 1.13 [0.81, 1.67] | 1.19 [0.81, 1.84] | 0.491 |
| HDL-Cholesterol, mmol/L | 1.37 ± 0.30 | 1.39 ± 0.30 | 0.539 |  | 1.37 ± 0.29 | 1.39 ± 0.31 | 0.331 |  | 1.38 ± 0.30 | 1.37 ± 0.27 | 0.829 |
| LDL-Cholesterol, mmol/L | 2.75 ± 0.76 | 2.68 ± 0.67 | 0.146 |  | 2.69 ± 0.69 | 2.81 ± 0.82 | 0.012 |  | 2.71 ± 0.73 | 2.82 ± 0.74 | 0.13 |
| Hypertension (%) | 163 (23.0) | 66 (19.1) | 0.178 |  | 170 (22.5) | 59 (19.7) | 0.365 |  | 202 (21.8) | 27 (21.1) | 0.943 |
| Diabetes (%) | 61 (8.8) | 20 (5.9) | 0.142 |  | 55 (7.5) | 26 (8.8) | 0.548 |  | 64 (7.1) | 17 (13.4) | 0.021 |
| Obstructive Sleep Apnea (%) | 6 (0.8) | 10 (2.9) | 0.01 |  | 7 (0.9) | 9 (3.0) | 0.02 |  | 16 (1.7) | 0 (0) | 0.24 |
| Sleep disturbance (%) | / | / | / |  | 191 (25.2) | 157 (52.2) | <0.001 |  | 306 (32.9) | 42 (32.8) | 1 |
| Nighttime sleep < 7 hours (%) | 144 (20.3) | 157 (45.1) | <0.001 |  | / | / | / |  | 263 (28.3) | 38 (29.7) | 0.821 |
| Daytime napping > 60 minutes (%) | 86 (12.1) | 42 (12.1) | 1 |  | 90 (11.9) | 38 (12.6) | 0.821 |  | / | / | / |
| Continuous variables are expressed as mean ± standard deviation or as median [IQR]. Categorical variables are expressed as number (percent). HDL, high-density lipid. LDL, low-density lipid. Hs-CRP, high sensitivity C- reactive protein. HbA1c, hemoglobin A1c. | | | | | | | | | | | |

**Table S2. Subgroup analysis of the association between sleep disturbance and elevated galectin-3.**

|  | Odd ratio (95% confidence interval) ^d^ | P-interaction |  |
| --- | --- | --- | --- |
| Age, years ^a^ |  | 0.15 |  |
| ≥ 50 | 1.14 (0.62, 2.10) |  |  |
| < 50 | 2.33 (1.18, 4.62) |  |  |
| Sex ^b^ |  | 0.07 |  |
| Male | 2.54 (1.28, 5.05) |  |  |
| Female | 1.11 (0.60, 2.04) |  |  |
| Obesity ^c^ |  | 0.98 |  |
| Yes | 1.81 (0.46, 7.11) |  |  |
| No | 1.58 (0.97, 2.58) |  |  |
| Abdominal obesity ^c^ |  | 0.65 |  |
| Yes | 1.76 (0.94, 3.28) |  |  |
| No | 1.44 (0.73, 2.88) |  |  |
| Hypertension ^c^ |  | 0.10 |  |
| Yes | 0.85 (0.35, 2.06) |  |  |
| No | 2.04 (1.18, 3.52) |  |  |
| Diabetes ^c^ |  | 0.21 |  |
| Yes | 0.41 (0.04, 4.08) |  |  |
| No | 1.78 (1.11, 2.86) |  |  |
| ^a^ adjusted for sex. ^b^ adjusted for age. ^c^ adjusted for age and sex. ^d^ With sleep disturbance vs. without sleep disturbance | | |  |
|  |  |  |  |

**Table S3. Association between sleep patterns and elevated galectin-3 according to different cutoffs**

|  | Odd ratio (95% confidence interval) | | |  |
| --- | --- | --- | --- | --- |
| Cutoffs ^d^ | Sleep disturbance ^a^ | Nighttime sleep duration ^b^ | Daytime napping duration ^c^ |  |
| The 50% quantile | 1.38 (1.04, 1.83) | 1.27 (0.95, 1.70) | 1.48 (0.98, 2.24) |  |
| The 60% quantile | 1.35 (1.02, 1.80) | 1.08 (0.80, 1.45) | 1.49 (0.98, 2.24) |  |
| The 70% quantile | 1.37 (1.02, 1.85) | 0.93 (0.68, 1.27) | 1.45 (0.95, 2.21) |  |
| The 80% quantile | 1.43 (1.03, 2.00) | 0.93 (0.65, 1.32) | 1.52 (0.97, 2.39) |  |
| The 90% quantile | 1.90 (1.23, 2.94) | 1.06 (0.67, 1.68) | 1.05 (0.57, 1.96) |  |
| ^a^ yes vs. no. ^b^ < 7 hours vs. ≥ 7 hours. ^c^ ≥ 60 minutes vs. < 60 minutes  ^d^ elevated galectin-3 was defined as the galectin-3 concentration equal or over the cutoff.  Model 3. adjusted for age, sex, current smoking, alcohol consumption, systolic blood pressure, Obstructive Sleep Apnea, body mass index, waist, fasting blood glucose, triglyceride, high density lipid cholesterol and high-sensitivity C- reactive protein. | | | |  |

**Table S4. Association between subtypes of sleep disturbance and elevated galectin-3**

|  | Odd ratio (95% confidence interval) |  |
| --- | --- | --- |
| Self-reported poor sleep quality | 1.33 (1.02, 1.74) |  |
| Difficulty in falling asleep | 1.93 (1.06, 3.51) |  |
| Frequently waking up during the night | 1.40 (0.86, 2.27) |  |
| Models were adjusted for age, sex, current smoking, alcohol consumption, systolic blood pressure, Obstructive Sleep Apnea, body mass index, waist, fasting blood glucose, triglyceride, high density lipid cholesterol and high-sensitivity C- reactive protein.  Elevated galectin-3 was defined as galectin-3 level > 65.1 ng/ml. | |  |
|  |  |  |
|  |  |  |

**Table S5. Sensitivity analysis: association between nighttime sleep duration and daytime napping duration with elevated galectin-3**

| Odd ratio (95% confidence interval) | | |
| --- | --- | --- |
|  | Nighttime sleep duration ^a^ | Daytime napping duration ^b^ |
| Model 1 | 1.21 (0.77, 1.92) | 1.16 (0.75, 1.81) |
| Model 2 | 1.28 (0.80, 2.05) | 1.00 (0.63, 1.58) |
| Model 3 | 1.34 (0.82, 2.18) | 0.90 (0.56, 1.45) |
| ^a^ < 8 hours vs. ≥ 8 hours. ^b^ ≥ 30 minutes vs. < 30 minutes  Model 1. crude model.  Model 2. adjusted for age and sex.  Model 3. adjusted for age, sex, current smoking, alcohol consumption, systolic blood pressure, Obstructive Sleep Apnea, body mass index, waist, fasting blood glucose, triglyceride, high density lipid cholesterol and high-sensitivity C- reactive protein.  Elevated galectin-3 was defined as galectin-3 level > 65.1 ng/ml. | | |

**Figure S1. Distribution of galectin-3 according to sex.**

**
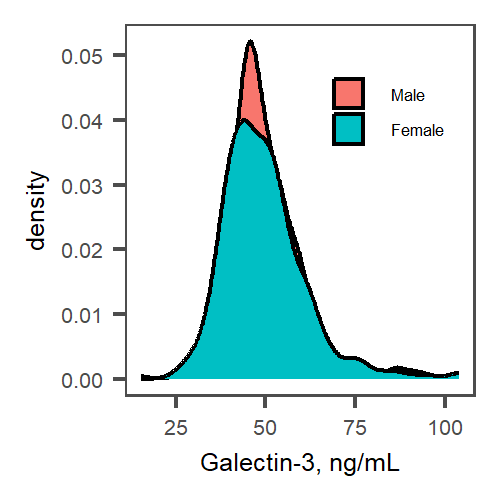
**
